# ASSOCIATION BETWEEN SUBJECTIVE AND OBJECTIVE COGNITIVE FUNCTION IN CHINA

**DOI:** 10.1093/geroni/igz038.742

**Published:** 2019-11-08

**Authors:** Bada Kang, Hanzhang Xu, Eleanor S McConnell, Bei Wu

**Affiliations:** 1 Duke University School of Nursing, Durham, North Carolina, United States; 2 Duke University Department of Community & Family Medicine, Durham, North Carolina, United States; 3 Duke University School of nursing Geriatric Research; Education and Clinical Center, Durham Department of Veterans Affairs Medical Center, Durham, North Carolina, United States; 4 New York University College of nursing, New York, New York, United States

## Abstract

Although subjective cognitive decline is considered as a potential symptomatic indicator of cognitive decline, little is known regarding the relationships in older adults in China. Using the World Health Organization Study on global AGEing and adult health (SAGE) Wave 1 data, we examined the association between subjective cognitive function, perceived memory decline, and objective cognitive function among adults aged 50 or older (N=13,367) in China. Objective cognitive function was measured by immediate and delayed recall test, digit span test, and verbal fluency test. Multivariate linear regression models were used to account for sociodemographic, psychosocial, and health-related factors. We found worse subjective cognitive function was associated with poorer working memory and verbal fluency. Greater perceived memory decline was also associated with poorer working memory but not with verbal fluency. Psychosocial factors including social cohesion and social support attenuated the relationships between subjective cognitive function, perceived memory decline, and objective cognitive performance.

